# Hsc70 Ameliorates the Vesicle Recycling Defects Caused by Excess α-Synuclein at Synapses

**DOI:** 10.1523/ENEURO.0448-19.2020

**Published:** 2020-01-28

**Authors:** Susan M. L. Banks, Audrey T. Medeiros, Molly McQuillan, David J. Busch, Ana Sofia Ibarraran-Viniegra, Rui Sousa, Eileen M. Lafer, Jennifer R. Morgan

**Affiliations:** 1 The Eugene Bell Center for Regenerative Biology and Tissue Engineering Marine Biological Laboratory, Woods Hole, MA 02543; 2 Department of Biomedical Engineering, The University of Texas at Austin, Austin, TX 78712; 3 Department of Biochemistry and Structural Biology and Center for Biomedical Neuroscience, the University of Texas Health Science Center at San Antonio, San Antonio, TX 79229

**Keywords:** auxilin, chaperone, clathrin, clathrin-coated vesicles, endocytosis, lamprey

## Abstract

α-Synuclein overexpression and aggregation are linked to Parkinson’s disease (PD), dementia with Lewy bodies (DLB), and several other neurodegenerative disorders. In addition to effects in the cell body, α-synuclein accumulation occurs at presynapses where the protein is normally localized. While it is generally agreed that excess α-synuclein impairs synaptic vesicle trafficking, the underlying mechanisms are unknown. We show here that acute introduction of excess human α-synuclein at a classic vertebrate synapse, the lamprey reticulospinal (RS) synapse, selectively impaired the uncoating of clathrin-coated vesicles (CCVs) during synaptic vesicle recycling, leading to an increase in endocytic intermediates and a severe depletion of synaptic vesicles. Furthermore, human α-synuclein and lamprey γ-synuclein both interact *in vitro* with Hsc70, the chaperone protein that uncoats CCVs at synapses. After introducing excess α-synuclein, Hsc70 availability was reduced at stimulated synapses, suggesting Hsc70 sequestration as a possible mechanism underlying the synaptic vesicle trafficking defects. In support of this hypothesis, increasing the levels of exogenous Hsc70 along with α-synuclein ameliorated the CCV uncoating and vesicle recycling defects. These experiments identify a reduction in Hsc70 availability at synapses, and consequently its function, as the mechanism by which α-synuclein induces synaptic vesicle recycling defects. To our knowledge, this is the first report of a viable chaperone-based strategy for reversing the synaptic vesicle trafficking defects associated with excess α-synuclein, which may be of value for improving synaptic function in PD and other synuclein-linked diseases.

## Abstract (precis)

α-Synuclein overexpression and aggregation are linked to Parkinson’s disease (PD), dementia with Lewy bodies (DLB), and several other neurodegenerative disorders. In addition to effects in the cell body, α-synuclein accumulation occurs at presynapses where the protein is normally localized. While it is generally agreed that excess α-synuclein impairs synaptic vesicle trafficking, the underlying mechanisms are unknown.

## Significance Statement

Synaptic defects caused by α-synuclein overexpression are linked to cognitive deficits in Parkinson’s disease (PD) and other diseases. However, the mechanisms by which excess α-synuclein impairs synaptic vesicle trafficking are unknown. Data presented here demonstrate that acute introduction of excess α-synuclein at a classical vertebrate synapse inhibits clathrin-coated vesicle (CCV) uncoating, leading to impaired vesicle recycling. Furthermore, increasing α-synuclein also reduced synaptic levels of Hsc70, the clathrin uncoating chaperone protein, indicating sequestration of Hsc70 as a possible underlying mechanism. Subsequently increasing exogenous Hsc70 restored CCV uncoating and vesicle recycling. This study identifies a novel molecular mechanism underlying the α-synuclein-induced synaptic defects and presents one viable strategy for reversing them.

## Introduction

α-Synuclein is a presynaptic protein whose aberrant aggregation causes neurodegeneration in Parkinson’s disease (PD), dementia with Lewy bodies (DLB), and several variants of Alzheimer’s disease (Singleton et al., 2003; Lee and Trojanowski, 2006; Cookson and van der Brug, 2008; Ingelsson, 2016). Inherited forms of PD are linked to multiplication of the α-synuclein gene (*SNCA*), as well as several point mutations (e.g., A30P, E46K, A53T), which result in atypical aggregation of α-synuclein protein throughout neurons (Singleton et al., 2003; Lee and Trojanowski, 2006). α-Synuclein aggregation causes synaptic dysfunction, mitochondrial damage, and axonal transport deficits (Ingelsson, 2016). Though the mechanisms of α-synuclein toxicity are beginning to emerge, particularly with respect to events in the soma, much less is known about how increased α-synuclein levels affect synapses where the protein normally functions.

Under physiologic conditions, α-synuclein participates in synaptic vesicle trafficking via its established roles in vesicle clustering/reclustering (Nemani et al., 2010; Atias et al., 2019), SNARE complex assembly (Burré et al., 2010), vesicle fusion (Bendor et al., 2013; Logan et al., 2017, Sulzer and Edwards, 2019), and synaptic vesicle trafficking/recycling (Cabin et al., 2002; Greten-Harrison et al., 2010; Vargas et al., 2014; Wang et al., 2014; Sun et al., 2019). In contrast, much less is known about the impacts of excess α-synuclein at synapses, which occurs during disease pathologies. Acutely increasing α-synuclein at several vertebrate synapses leads to an inhibition of synaptic vesicle recycling (Busch et al., 2014; Xu et al., 2016; Eguchi et al., 2017). Overexpression of α-synuclein in animal models of PD induces the formation of small oligomers, microaggregates, and filaments at synapses, following a similar pathologic cascade that occurs in other neuronal compartments (Scott et al., 2010; Volpicelli-Daley et al., 2011; Spinelli et al., 2014). This leads to a loss of several critical presynaptic proteins within boutons, which has also been observed in brains of DLB patients (Scott et al., 2010). Perhaps as a consequence, synaptic aggregation of α-synuclein is highly correlated with cognitive deficits in some DLB and PD patients (Kramer and Schulz-Schaeffer, 2007; Schulz-Schaeffer, 2010; Ingelsson, 2016). Despite these indications that excess and/or aggregated α-synuclein negatively impacts presynaptic functions, the underlying mechanisms are unknown.

Lamprey giant reticulospinal (RS) synapses provide an excellent model for assessing how excess α-synuclein affects vertebrate synapses. RS synapses are ideal for these studies because they are amenable to acute perturbations of presynaptic processes (Brodin and Shupliakov, 2006; Walsh et al., 2018), thus permitting a direct evaluation of the effects of excess α-synuclein without inducing molecular compensation that occurs after overexpression (Nemani et al., 2010; Scott et al., 2010). Furthermore, the large size of vesicle clusters at RS synapses facilitates detailed ultrastructural analyses of synaptic vesicle trafficking events, allowing us to determine the underlying mechanisms (Morgan et al., 2004, 2013; Busch et al., 2014). We previously reported that acute introduction of excess human α-synuclein inhibited synaptic vesicle recycling at lamprey synapses, and the results were consistent with effects on both clathrin-mediated endocytosis (CME) and possibly compensatory bulk endocytosis (Busch et al., 2014; Medeiros et al., 2017, 2018). Similarly, excess α-synuclein also inhibited vesicle endocytosis at mammalian calyx of Held synapses (Xu et al., 2016; Eguchi et al., 2017).

We report here a mechanism by which excess α-synuclein induces synaptic vesicle recycling defects and present a novel strategy for ameliorating these defects. When introduced acutely, α-synuclein selectively impaired CCV uncoating during synaptic vesicle recycling, leading to a depletion of the synaptic vesicle cluster (Medeiros et al., 2017). We further show that human α-synuclein, lamprey γ-synuclein, and several PD-linked mutants directly associate *in vitro* with Hsc70, the chaperone protein that uncoats CCVs at synapses, thus identifying an interaction that may affect synapses *in vivo*. Indeed, excess α-synuclein reduced Hsc70 availability at stimulated synapses, suggesting Hsc70 sequestration as a possible mechanism underlying the synaptic defects. Consequently, co-injection of exogenous Hsc70 and α-synuclein ameliorated the synaptic vesicle trafficking defects. Thus, Hsc70 is an *in vivo* target of excess α-synuclein at synapses, and increasing Hsc70 function reverses the deleterious impacts.

## Materials and Methods

### Recombinant proteins

Cloning of recombinant GST-tagged human α-synuclein and His-tagged bovine Hsc70 used for biochemistry experiments was as described previously (Wilbanks et al., 1995; Busch and Morgan, 2012; Busch et al., 2014; Sousa et al., 2016). Recombinant proteins were expressed in BL21-CodonPlus (DE3)-RILP Competent Cells (Agilent Technologies) and purified using Glutathione Sepharose 4B Media (GE Healthcare) or Ni-NTA resin (Thermo Fisher Scientific). Untagged human α-synuclein used in the microinjection experiments was obtained from rPeptide.

### Acute perturbations and electron microscopy

All animal procedures were approved by the Institutional Animal Care and Use Committee at the MBL in accordance with standards set by the National Institutes of Health. Lampreys (*Petromyzon marinus*; 11–13 cm; five to seven years old of either sex) were anesthetized in 0.1 g/l MS-222 (Western Chemical Inc.). Spinal cord pieces (2–3 cm) were dissected and pinned ventral side up in a Sylgard-lined dish. Axonal microinjections were performed as described in Walsh et al., (2018). First, human α-synuclein was diluted in lamprey internal solution (180 mM KCl and 10 mM HEPES K^+^; pH 7.4) to a pipet concentration of 130–160 μM. In some experiments, recombinant bovine Hsc70 (27 μM) was included in the injection pipet either alone or together with α-synuclein. Proteins were then loaded into glass microelectrodes (20–25 MΩ) and microinjected into giant RS axons using small pulses of N_2_ (5–20 ms, 30–50 psi, 0.2 Hz) delivered through a picospritzer. Fluorescein (10 kDa) or tetramethylrhodamine (70 kDa) dextrans (100 μM; Thermo Fisher), approximating the molecular weights of α-synuclein and Hsc70, respectively, were included in the pipets and co-injected to visualize the proteins’ diffusion rates in the axons. Axonal injections resulted in a 10–20× dilution of the proteins. Thus, the final axonal concentration of exogenous human α-synuclein was estimated to be ∼7–13 μM, and the final concentration of exogenous bovine Hsc70 was ∼1–3 μM. Axons were subsequently stimulated with action potentials (20 Hz, 5 min) using current injections (30–60 nA; 1 ms) to induce synaptic vesicle exocytosis/endocytosis.

**Figure 1.**
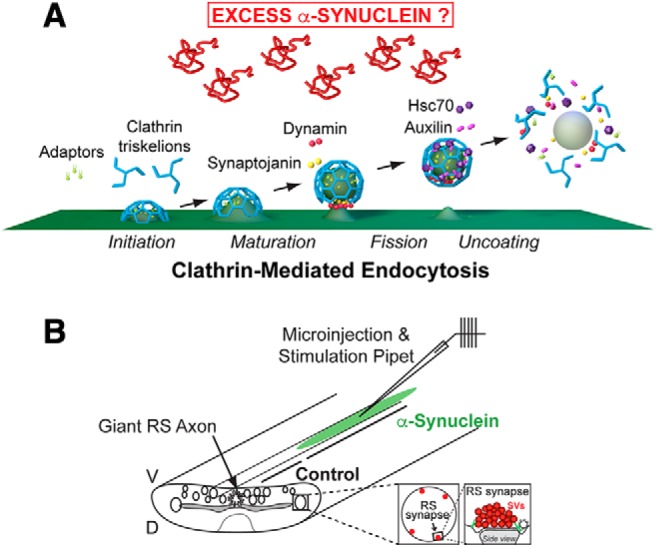
Project goal and lamprey model. ***A***, Diagram showing the major stages of clathrin-mediated synaptic vesicle endocytosis and several molecular players. The goal of the study is to determine how excess α-synuclein affects this process and the underlying molecular mechanisms. Graphics generated by Jack Cook and Tim Silva (Woods Hole Oceanographic Institution) using Cinema 4D. ***B***, Diagram of the lamprey spinal cord showing microinjection strategy and location of RS synapses. All electron microscopy experiments were internally controlled such that the control synapses were taken from a region of the injected axon beyond which the reagents (e.g., α-synuclein) had diffused. D = dorsal; V = ventral.

Spinal cords were fixed immediately after stimulation (3% glutaraldehyde, 2% paraformaldehyde in 0.1 M Na cacodylate; pH 7.4), processed for EM, sectioned at 70 nm, and counterstained with uranyl acetate and lead citrate, as described previously (Morgan et al., 2013; Busch et al., 2014; Walsh et al., 2018). Images were obtained at 37,000× or 59,000× magnification using a JEOL JEM 200CX electron microscope. We collected EM data on *n* = 22–33 synapses from at least two axons from two lampreys per condition and confirmed that the phenotypes reported were consistent between axons/animals. Images were collected at distances surrounding the injection site (20–150 μm) where the protein concentration was measurable based on the diffusion of the co-injected fluorescent dye (i.e., the experimental condition), as well as distances farther from the injection site (150–700 μm) where no protein had diffused (i.e., the controls). Thus, each EM experiment was internally controlled, as shown in Figure 1*B*, which is necessary due to the variability in the sizes of synaptic vesicle clusters between axons and animals. 3D reconstructions were generated from five serial electron micrographs using Reconstruct software (Fiala, 2005), as described previously (Busch et al., 2014; Medeiros et al., 2017). One image per synapse, taken at or near the center of the active zone, was selected for morphometric analysis. A researcher blinded to the experimental conditions performed the morphometric analyses on all synaptic membranes within a 1-μm radius of the active zone using FIJI 2.0.0 (Morgan et al., 2004, 2013; Busch et al., 2014). These included synaptic vesicles, plasma membrane, cisternae, and Clathrin-coated pits (CCPs) and clathrin-coated vesicles (CCVs). Synaptic vesicles were defined as small, clear round vesicles <100 nm in diameter, while “cisternae” were defined as larger vesicles that were >100 nm in diameter, as in our previous studies (Busch et al., 2014; Medeiros et al., 2017). Plasma membrane evaginations were determined by drawing a straight line from the edge of the active zone to the nearest position on the axolemma, on both sides of the synapse, and then measuring the curved distance between these points; the mean value per synapse was recorded. CCPs and CCVs were staged as described previously (Morgan et al., 2004). In addition, the SV distribution was determined using a script written in Python (https://github.com/audreytmedeiros/Morgan-Lab), which measured the distance from the center of each SV to the nearest point on the active zone. After obtaining measurements for each organelle, a total membrane analysis was performed on each synapse to determine how synaptic membranes were redistributed with each perturbation. Here, SV and CCP/V membrane areas were calculated by multiplying the surface area of a sphere (4πr^2^) by the number of each type of vesicle at each synapse. Plasma membrane and cisternae areas were obtained by multiplying the length of membrane evaginations and summed cisternae perimeters, respectively, by the section thickness (70 nm). Graphing and statistical analyses, including Student’s *t* tests and ANOVA, were performed in Origin 7.0 (OriginLab Corp). Data were reported as the mean value per section per synapse.

### GST pull downs

Protein lysates from rat brains or lamprey central nervous system (CNS) (brains and spinal cords) were prepared in HKET buffer (25 mM HEPES K^+^, 150 mM KCl, 1 mM EDTA, and 1% Triton X-100; pH 7.4) containing protease inhibitors. GST pull downs were performed using 50–100 μg of GST-tagged proteins and either 1–5 mg of rat brain or lamprey CNS extracts or 100 μg of purified recombinant proteins. After performing the pull downs, the bound proteins were run on 10% SDS-PAGE gels and transferred to nitrocellulose membranes. For Western blotting, antibodies were diluted in TBST (20 mM Tris pH 7.6, 150 mM NaCl, and 0.1% Tween 20) with 1% dry milk or 5% BSA. Primary antibodies were used at 1:1000 and included: mouse monoclonal anti-β1/β2-adaptins (clone 100/1; Sigma), mouse monoclonal anti-clathrin heavy chain (clone 23; BD Biosciences), mouse monoclonal anti-dynamin (clone 41; BD Biosciences), mouse monoclonal anti-synaptojanin-1 (clone 26; BD Biosciences), mouse monoclonal anti-auxilin (gift from Ernst Ungewickell; Ungewickell et al., 1995), rabbit polyclonal anti-Hsc70 (ARP48445; Aviva Systems Biology), and rabbit polyclonal anti-Hsc70 (SPA-816; Enzo Life Sciences). Secondary antibodies were used at 1:1000–1:4000 and included HRP-conjugated goat anti-rabbit, anti-mouse, and anti-rat IgGs (H + L; Thermo Scientific). The Hsc70 antibodies were validated in lamprey by the appearance of a single protein band of the correct molecular weight matching that in rat brain and by the elimination of that band in secondary only control experiments. Protein bands were detected using Pierce ECL Western blotting substrate (Thermo Scientific). Band intensities were measured using FIJI 2.0.0 software and statistically analyzed in Prism 8.0 (GraphPad Software, Inc.). Data shown in biochemistry figures indicate mean ± SEM from *n* = 3–5 experiments.

### Clathrin uncoating assays

Clathrin cages were assembled with 1 μM recombinant bovine brain clathrin and 0.1 μM auxilin, as described in Sousa et al. (2016). CCVs were freshly purified from bovine brains as described previously (Keen et al., 1979; Nandi et al., 1982). To visualize the clathrin cages and CCVs, freshly glow-discharged copper grids (EM Sciences) were floated onto a drop of each sample for 5 min, followed by six washes in distilled H_2_O, counterstaining in 1% uranyl acetate for 3 min in the dark. After drying, the grids were imaged on a JEOL JEM 200CX electron microscope at 100 kV using 100,000× magnification. Clathrin disassembly from clathrin cages and purified CCVs was measure *in vitro* by light-scattering experiments conducted in an Applied Photosystems stopped-flow fluorometer with excitation/emission wavelengths of 395 nm as described in Sousa et al. (2016). Briefly, clathrin cages or purified CCVs corresponding to 0.3 μM clathrin heavy chain with 1 mM ATP and 0.45 μM auxilin in 20 mM imidazole, pH 6.8, 10 mM (NH_4_)_2_SO_4_, 25 mM KCl, and 2 mM MgAc_2_ were reacted with an equal volume of 4 μM Hsc70 in the same buffer. Background scattering determined from reactions without cages or CCVs was subtracted from measured scattering values, which were normalized by dividing by the starting scattering value so that the initial scattering in all reactions was 1.0. In some reactions 20 μM recombinant human α-synuclein (rPeptide) was added to the Hsc70 syringe. Various components were also omitted when indicated as controls. Data were plotted using Origin 7.0 software.

### Immunofluorescence (IF) at lamprey synapses

Recombinant human α-synuclein was microinjected into giant RS axons as described above for the EM experiments, after which spinal cords were stimulated using high K^+^ (50 mM) Ringer for 10 min (Wickelgren et al., 1985). In some experiments, spinal cords were stimulated using action potentials (20 Hz, 5 min). Following stimulation, spinal cords were fixed in 4% paraformaldehyde in 0.1 M PBS, pH 7.4, for 3 h, washed in 0.1 M PBS, and incubated for 1 h in blocking buffer (10% normal goat serum; Thermo Fisher Scientific) containing 0.3% Triton X-100. Primary antibody incubations were overnight at 4°C, followed by 5 × 1 h washes in wash buffer (20 mM Na phosphate buffer, 450 mM NaCl, and 0.3% Triton X-100; pH 7.4). Primary antibodies included: mouse monoclonal anti-SV2 antibody, which was deposited to DSHB by K. M. Buckley (1:100; DSHB; Buckley and Kelly, 1985), rabbit polyclonal anti-Hsc70 (ARP48445; Aviva Systems Biology, Corp.), and rat monoclonal anti-Hsc70 (SPA-815; Enzo Life Sciences). Secondary antibodies used were Alexa Fluor 594 goat anti-mouse IgG (H + L; 1:200), Alexa Fluor 488 goat anti-rabbit IgG (H + L; ThermoFisher), or DyLight 488 goat anti-rat IgG (H + L; 1:100; Thermo Fisher). After immunostaining, synapses were imaged within intact whole mounted spinal cords using a Zeiss LSM510 Meta confocal on an Axioskop 2FS microscope. Images were acquired using a Zeiss 40×, 0.8 NA Achroplan objective with 3× optical zoom. All analyses on synapses were performed in FIJI 2.0.0 as follows. Giant RS synapses were first identified using the SV2 labeling, and then the associated Hsc70 puncta (≥2× background intensity) that were located on and around the synapses were identified. Those that were overlapping or touching the SV2 puncta were considered to be associated with the synaptic vesicle cluster while those within a 1-μm radius of the synapses were located within the endocytic periactive zone. The percentage of synapses containing Hsc70 puncta within each axon, as well as the average number of Hsc70 puncta per synapse, was calculated. All graphing and statistical analyses were performed in GraphPad Prism 8.0 software, including outlier and normality tests (Shapiro–Wilk; D’Agostino and Pearson), as well as statistical comparisons using Student’s *t* test or ANOVA.

## Results

### Excess α-synuclein impairs clathrin uncoating *in vivo* during synaptic vesicle recycling

We previously reported that acute introduction of excess human α-synuclein to lamprey RS synapses caused a reduction in the number of synaptic vesicles, which was compensated by an increase in the size of plasma membrane evaginations, as well as greater numbers of irregular membranous cisternae and clathrin-coated structures [i.e., the total number of CCPs and CCVs combined; Busch et al., 2014]. Many of the cisternae originated from the plasma membrane and had CCPs budding from them, suggesting that they were plasma membrane extensions and/or bulk endosomes derived through compensatory endocytosis, though we cannot rule out an effect on recycling endosomes (Chanaday et al., 2019). The phenotype reported is consistent with an impairment of synaptic vesicle recycling via inhibition of CME and possibly bulk endocytosis. Because CME is a predominant mechanism for locally recycling synaptic vesicles at many synapses (Heuser and Reese, 1973; Granseth et al., 2006; Heerssen et al., 2008; Walsh et al., 2018), and because clathrin-mediated vesicle budding is also critical for other modes of SV recycling such as ultrafast and bulk endocytosis (Watanabe et al., 2014; Gan and Watanabe, 2018; Chanaday et al., 2019), we set out to determine in this study exactly how excess α-synuclein impacts clathrin-mediated processes at synapses and to identify the underlying mechanisms.

To identify how excess α-synuclein impairs CME, we first needed to determine which stage or stages were preferentially affected. Briefly, clathrin-mediated vesicle recycling is initiated when clathrin adaptors, AP180 and AP2, recruit clathrin triskelia to the plasma membrane, promoting their assembly into coats (Fig. 1*A*; Morgan et al., 1999; Saheki and De Camilli, 2012). After maturation of the CCP, vesicle fission occurs through the actions of dynamin, a large GTPase that is abundant at synapses (Takei et al., 1995; Antonny et al., 2016). Synaptojanin is recruited to the neck of the CCP to assist during fission and subsequently in CCV uncoating (Cremona et al., 1999; Chang-Ileto et al., 2011). After vesicle fission is complete, the free CCVs are uncoated by the actions of the chaperone protein Hsc70 and its co-chaperone, auxilin (Ungewickell et al., 1995; Morgan et al., 2001). Uncoated vesicles are then refilled with neurotransmitter molecules (Farsi et al., 2018) and returned to the vesicle cluster for subsequent bouts of exocytosis.

**Figure 2.**
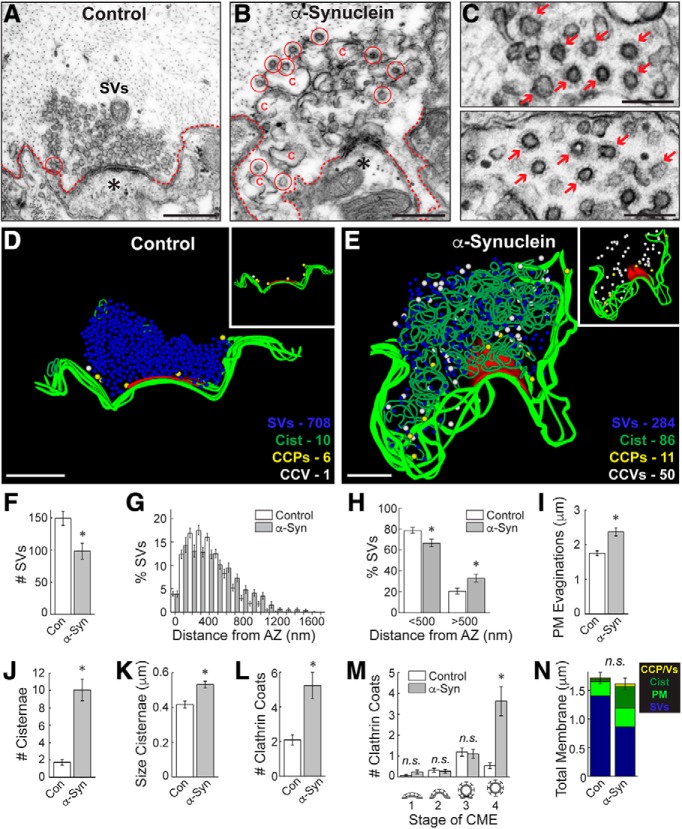
Excess α-synuclein impairs CCV uncoating during synaptic vesicle recycling. ***A***, ***B***, Electron micrographs of untreated, control lamprey synapses stimulated at 20 Hz for 5 min. After stimulation, control synapses have large synaptic vesicle (SV) clusters, shallow plasma membrane (PM) evaginations (dotted lines), and only a few CCP/Vs (circles). In contrast, synapses treated with excess human α-synuclein had smaller SV clusters, larger PM evaginations, and greater numbers of CCP/Vs, indicative of a vesicle recycling defect. Asterisks mark postsynaptic spines. Scale bars = 500 nm. ***C***, Insets show clusters of free CCVs at synapses after treatment with α-synuclein (arrows). Scale bars = 200 nm. ***D***, ***E***, 3D reconstructions comparing control and α-synuclein treated synapses. α-Synuclein caused severe endocytic defects, including a striking increase in CCVs (white spheres) and cisternae (dark green traces). Insets show the distributions of CCPs (yellow spheres) and CCVs (white spheres). While CCP/Vs at control synapses are sparse and localized near the plasma membrane at control synapses (***D***, inset), α-synuclein treated synapses exhibited large numbers of free CCVs throughout the synaptic area with little change in CCPs (***E***, inset). Active zone is shown in red. Scale bars = 500 nm. ***F–N***, The SV recycling defect induced by α-synuclein is demonstrated by a loss of SVs (***F***) and synaptic vesicle dispersion (***G***, ***H***), which was compensated by larger PM evaginations (***I***) and greater numbers/size of cisternae (***J***, ***K***) as well as CCP/Vs (***L***). The selective increase in CCVs (stage 4) indicates a clathrin uncoating defect (***M***). Total membrane analysis reveals a redistribution of synaptic membranes reflecting the endocytic defect. Bars represent mean ± SEM (per section, per synapse) from *n* = 30–33 synapses, *n* = 2 axons, *n* = 2 animals/condition. Asterisks denote significance (*p* < 0.05) by Student’s *t* test (***F***, ***I–L***, ***N***) or ANOVA (***H***, ***M***). n.s. = not significant by ANOVA.

As in our prior studies, we microinjected recombinant monomeric human α-synuclein into lamprey giant axons, thereby delivering the protein directly to presynapses (Fig. 1*B*). The axons were subsequently stimulated (20 Hz, 5 min), fixed, and processed for standard transmission electron microscopy (Walsh et al., 2018). After injection, the final axonal concentration of α-synuclein was estimated to be ∼7–13 μM, which is approximately two to four times greater than measurements of endogenous α-synuclein at synapses and commensurate with overexpression levels in mammalian PD models and human patients (Singleton et al., 2003; Miller et al., 2004; Nemani et al., 2010; Scott et al., 2010; Westphal and Chandra, 2013). Images of untreated control synapses were collected from the same axon but from regions beyond where the α-synuclein protein had diffused (Fig. 1*B*), thus providing an internal control for each experiment, which is important because of the natural variability in the sizes of synaptic vesicle clusters between axons and animals. At control synapses, local synaptic vesicle recycling was efficient enough to maintain a large synaptic vesicle cluster, and very few CCP/Vs were observed (Fig. 2*A*,*D*). In contrast, after injection of α-synuclein, synapses were dramatically altered due to deficits in synaptic vesicle recycling. Specifically, synapses treated with α-synuclein exhibited fewer synaptic vesicles, expanded plasma membrane evaginations, increased numbers of large atypical cisternae, and abundant clathrin-coated structures (Fig. 2*B*,*E*), as previously reported (Busch et al., 2014; Medeiros et al., 2017). Cisternae were classified as any irregular-shaped vesicles with a diameter >100 nm (Busch et al., 2014; Medeiros et al., 2017). These cisternae often had CCPs budding from them and could sometimes be traced back to the plasma membrane (Busch et al., 2014). Strikingly, when we further examined the clathrin-coated structures, we observed atypical clusters of free CCVs at synapses treated with excess α-synuclein, a phenotype typically associated with defective CCV uncoating (Fig. 2*C*, arrows; Cremona et al., 1999; Morgan et al., 2001). 3D reconstructions generated from serial micrographs revealed the gross alterations in synaptic structure caused by α-synuclein (Fig. 2*D*,*E*). Whereas control synapses exhibited only a few CCPs and CCVs close to the plasma membrane, the α-synuclein treated synapses exhibited dozens of free CCVs that were dispersed throughout the synaptic area (Fig. 2*D*,*E*, insets).

We performed a quantitative morphometric analysis of all synaptic membranes within 1 μm of the active zone and confirmed that excess α-synuclein impaired synaptic vesicle recycling, as demonstrated by a loss of synaptic vesicles (Fig. 2*F*) that was compensated by larger plasma membrane evaginations (Fig. 2*I*; synaptic vesicles, control: 150 ± 11 SVs/section, *n* = 31 synapses, *n* = 2 axons; α-Synuclein: 98 ± 12 SVs; *n* = 30 synapses, *n* = 2 axons; Student’s *t* test; *p* < 0.005; plasma membrane, control: 1.75 ± 0.07 μm, *n* = 32 synapses, *n* = 2 axons; α-Synuclein: 2.37 ± 0.12 μm; *n* = 30 synapses, *n* = 2 axons; Student’s *t* test; *p* < 0.00005). The remaining synaptic vesicles were of normal size (diameter; control: 54.7 ± 0.6 nm, *n* = 200 SVs, *n* = 10 synapses; α-Synuclein: 52.9 ± 0.9 nm; *n* = 200 SVs, *n* = 10 synapses; Student’s *t* test; *p* = 0.10). However, the SVs were slightly more dispersed (Fig. 2*G*) and redistributed to longer distances from the active zone after treatment with α-synuclein (Fig. 2*H*; <500 nm, control: 78.87 ± 2.81%; α-Synuclein: 66.72 ± 3.71%; >500 nm, control: 20.81 ± 2.80%; α-Synuclein: 33.28 ± 3.71%; *n* = 30–32 synapses, *n* = 2 axons; ANOVA *p* < 0.0001; Tukey’s *post hoc p* < 0.05), which is consistent with the phenotype reported in hippocampal synapses from transgenic mice overexpressing human α-synuclein (Nemani et al., 2010). Corroborating the vesicle recycling defect, the number and the size (perimeter) of the cisternae were also significantly increased (Fig. 2*J*,*K*; #cisternae, control: 1.7 ± 0.3 cisternae, *n* = 32 synapses, *n* = 2 axons; α-Synuclein: 10.1 ± 1.2 cisternae; *n* = 30 synapses, *n* = 2 axons; Student’s *t* test; *p* < 0.00001; size cisternae, control: 0.42 ± 0.02 μm, *n* = 55 cisternae, *n* = 32 synapses; α-Synuclein: 0.53 ± 0.02 μm; *n* = 302 cisternae, *n* = 30 synapses; Student’s *t* test; *p* < 0.05). Supporting effects on CME, the total numbers of clathrin coats (CCPs+CCVs) were increased 2- to 3-fold (Fig. 2*L*; #clathrin coats, control: 2.1 ± 0.3 coats, *n* = 32 synapses, *n* = 2 axons; α-Synuclein: 5.2 ± 0.8 coats, *n* = 30 synapses, *n* = 2 axons; Student’s *t* test; *p* < 0.0005). Further dissection of the stages of CME revealed that α-synuclein selectively increased the number of free CCVs (stage 4) without significantly altering the earlier stages of CCP formation (stages 1–3; Fig. 2*M*; stage 1, control: 0.06 ± 0.04 CCPs/section; α-Synuclein: 0.23 ± 0.09 CCPs; stage 2, control: 0.31 ± 0.09 CCPs, α-Synuclein: 0.27 ± 0.10 CCPs; stage 3, control: 1.19 ± 0.20 CCPs; α-Syn: 1.10 ± 0.22 CCPs; stage 4, control: 0.53 ± 0.14 CCVs, α-Synuclein: 3.63 ± 0.70 CCVs; *n* = 30–32 synapses, *n* = 2 axons; ANOVA *p* < 0.0000005, Tukey’s *post hoc*). A total membrane analysis shows that the loss of synaptic vesicle membrane area was compensated by an expansion of the plasma membrane, cisternae, and CCP/Vs (Fig. 2*N*; control 1.7 ± 0.1 μm^2^; α-Syn: 1.6 ± 0.1 μm^2^; *n* = 30–32 synapses; Student’s *t* test; *p* = 0.47). Taken together, these data indicate that excess α-synuclein impairs clathrin-mediated synaptic vesicle recycling with selective effects on CCV uncoating (Medeiros et al., 2017, 2018). The larger plasma membrane evaginations and increased cisternae suggest additional effects of α-synuclein on initiation of CME (Vargas et al., 2014), although this could also be an indirect consequence of trapping limiting amounts of clathrin and coat proteins within the uncoated CCVs (Morgan et al., 2001).

### α-Synuclein interacts with Hsc70, the uncoating ATPase at synapses

**Figure 3.**
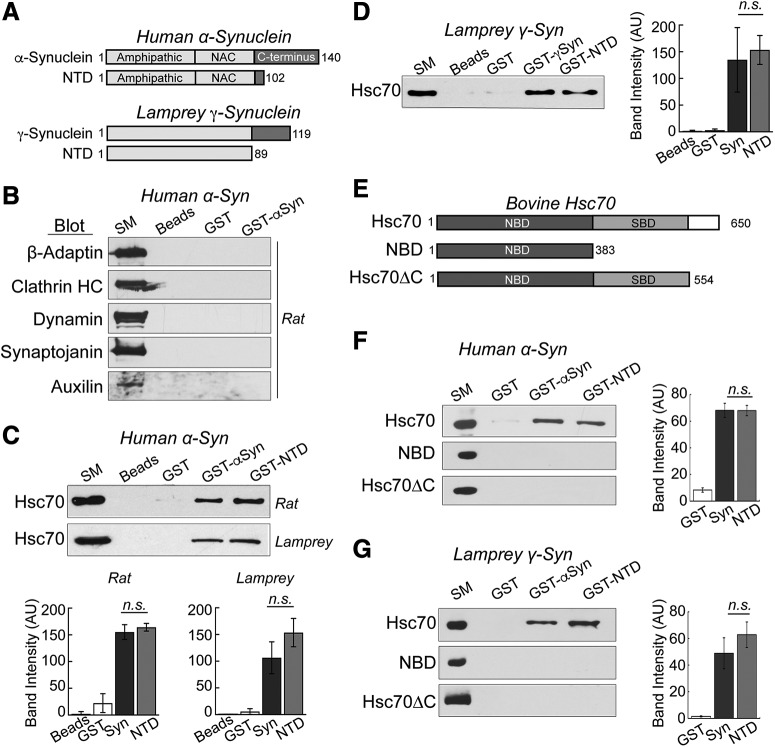
α-Synuclein interacts directly with Hsc70, the chaperone protein that uncoats CCVs during synaptic vesicle recycling. ***A***, Domain diagrams of full-length human α-synuclein, lamprey γ-synuclein and their respective NTDs. ***B***, GST pull downs from rat brain lysates revealed no detectable interactions between α-synuclein and several major components of CME including AP2, clathrin, dynamin, synaptojanin, and auxilin. SM = starting material. Blots shown are representative of *n* = 3 experiments. ***C***, In contrast, human α-synuclein and its NTD pulled down Hsc70 from rat brain and lamprey CNS lysates. ***D***, Similarly, lamprey γ-synuclein pulled down Hsc70 from rat brain lysates, demonstrating conservation of the interaction. ***E***, Domain diagrams of bovine Hsc70 and several truncations used in the experiments. ***F***, ***G***, In direct binding assays, both human α-synuclein and lamprey γ-synuclein, and their NTDs, pulled down recombinant bovine Hsc70. No interactions were detected with either NBD or Hsc70ΔC, indicating a role for the C terminus. In panels ***C***, ***D***, ***F***, ***G***, bars represent mean ± SEM from *n* = 3 independent experiments. n.s. = not significant by ANOVA (*p* > 0.05).

To determine how α-synuclein impairs clathrin uncoating, we next tested for possible interactions between α-synuclein and several key players that mediate CME at synapses, including those involved in the clathrin uncoating process (Fig. 1*A*; Saheki and De Camilli, 2012). Human α-synuclein consists of a highly conserved N-terminal domain (NTD; amino acids (a.a.) 1–95), which folds into an amphipathic α-helix on interaction with small vesicles and includes the non-Aβ component (NAC) domain (a.a. 61–95), followed by a less-structured acidic C-terminal domain (a.a. 96–140; Fig. 3*A*). GST-tagged human α-synuclein was used in pull-down experiments to test for any binding partners isolated from rat brain protein extracts. In the GST pull downs, no interactions were observed between α-synuclein and β-adaptin (an AP2 subunit), clathrin heavy chain, dynamin, synaptojanin, or auxilin (Fig. 3*B*). In contrast, full-length α-synuclein, as well as a truncation containing the highly conserved NTD (a.a. 1–102), selectively pulled down Hsc70, the chaperone protein that uncoats CCVs at synapses [Fig. 3*C*; beads: 2.1 ± 2.5 arbitrary units (AU); GST: 22.3 ± 10.2 AU; GST-αSyn: 155.2 ± 8.0 AU; GST-NTD: 164.2 ± 4.1 AU; *n* = 3; ANOVA *p* < 0.0001; Tukey’s *post hoc*]. Previous studies also reported an interaction between Hsc70 and α-synuclein with two identified binding regions within the first 102 amino acids (Pemberton et al., 2011; Redeker et al., 2012). Demonstrating conservation of the interaction, GST-α-synuclein and NTD also pulled down endogenous Hsc70 from lamprey brain and spinal cord extracts (Fig. 3*C*, bottom; beads: 0.3 ± 0.6 AU; GST: 5.7 ± 3.1 AU; GST-αSyn: 106.4 ± 17.2 AU; GST-NTD: 153.5 ± 15.3 AU; *n* = 3; ANOVA *p* < 0.0001, Tukey’s *post hoc*). We repeated the pull downs using GST-tagged lamprey γ-synuclein, which is the most highly expressed synuclein isoform in the lamprey giant RS neurons (Busch and Morgan, 2012). Full-length lamprey γ-synuclein is 56% identical and 63% similar to human α-synuclein, and its α-helical NTD is 67% identical and 90% similar to the corresponding region of human α-synuclein (Fig. 3*A*, bottom; Busch and Morgan, 2012; Busch et al., 2014). Further corroborating this conserved interaction, lamprey γ-synuclein and its NTD also pulled down Hsc70 from rat brain extracts (Fig. 3*D*; beads: 1.4 ± 0.9 AU; GST: 2.9 ± 1.3 AU; GST-γSyn: 134.9 ± 34.9 AU; GST-NTD: 153.3 ± 15.6 AU; *n* = 3; ANOVA *p* < 0.001; Tukey’s *post hoc*).

To determine whether the Hsc70/α-synuclein interaction is direct and to further map it, the GST pull downs were repeated using purified, recombinant bovine Hsc70. Hsc70 consists of a highly conserved N-terminal nucleotide-binding domain (NBD), and a substrate-binding domain (SBD) with a more variable 10 kDa C-terminal region (Fig. 3*E*). While the affinity of Hsc70 for typical client polypeptides is normally modulated by nucleotides (ATP/ADP), we performed the pull downs without nucleotides because prior studies reported more effective binding and sequestration of α-synuclein by Hsc70 in fibrillation assays under nucleotide-free conditions (Pemberton et al., 2011; Redeker et al., 2012). Under these conditions, GST-tagged human α-synuclein and its NTD pulled down bovine Hsc70, indicating a direct interaction (Fig. 3*F*, top; GST: 8.3 ± 1.6 AU; GST-Syn: 68.1 ± 5.3 AU; GST-NTD: 67.9 ± 3.9 AU; *n* = 3; ANOVA *p* = 5 × 10^−5^; Tukey’s *post hoc*). Similarly, lamprey γ-synuclein and its NTD also pulled down bovine Hsc70 in these direct binding assays (Fig. 3*G*, top; GST: 1.5 ± 0.4 AU; GST-Syn: 48.9 ± 11.6 AU; GST-NTD: 62.8 ± 9.6 AU; *n* = 3; ANOVA *p* = 0.006; Tukey’s *post hoc*). To further map the interaction, we additionally tested the binding of α-synuclein to several Hsc70 truncation mutants. One truncation consisted of only the NBD (a.a. 1–383), and the other contained the NBD and SBD but lacked the C-terminal 10 kDa region (Hsc70ΔC; a.a. 1–554; Fig. 3*E*; Flaherty et al., 1990; Wilbanks et al., 1995). Both truncation mutants are well expressed, folded, stable, soluble, and exhibit ATP and ADP binding affinities and ATP hydrolysis activity like full length Hsc70 (Wilbanks et al., 1995; Zhang and Zuiderweg, 2004; Jiang et al., 2005). Neither human α-synuclein, lamprey γ-synuclein, nor the NTDs pulled down the Hsc70 truncation mutants that were missing the C-terminal fragments, indicating a role for the C terminus in mediating or stabilizing the interaction (Fig. 3*F*,*G*, middle and bottom). Taken together, these data indicate that the N terminus of α-synuclein interacts directly with Hsc70 via an interaction that is conserved between synuclein orthologs and disrupted by the deletion of the C terminus of Hsc70.

**Figure 4.**
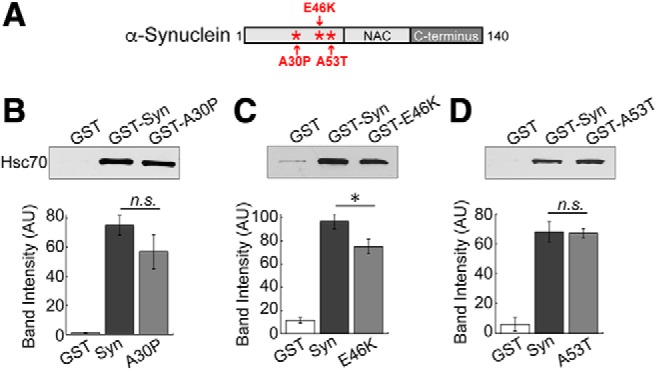
PD-associated α-synuclein mutants also interact directly with Hsc70. ***A***, Diagram showing the locations of PD-linked point mutations A30P, E46K, and A53T, which occur within the alpha helical NTD of α-synuclein. ***B–D***, In direct binding assays, GST-tagged A30P, E46K, and A53T all pulled down Hsc70 to a similar degree as wild-type α-synuclein. Only binding to E46K was slightly reduced. Bars represent mean ± SEM from *n* = 3–5 experiments. Asterisk indicates significance (*p* = 0.04); n.s. = not significant by ANOVA.

We repeated the pull downs using the α-synuclein point mutants that are linked to PD: A30P, E46K, and A53T (Fig. 4*A*). All three mutants bound directly to bovine Hsc70 (Fig. 4*B–D*). While A30P and A53T did not exhibit any statistically significant differences in binding efficacy, as compared to wild-type α-synuclein, E46K binding to Hsc70 was slightly reduced (Fig. 4*B–D*; GST: 1.1 ± 0.3 AU; GST-Syn: 75.0 ± 6.9 AU; GST-A30P: 57.0 ± 11.8 AU; *n* = 5; ANOVA *p* < 0.0001; Tukey’s *post hoc*; GST: 11.5 ± 2.3 AU; GST-Syn: 97.8 ± 5.5 AU; GST-E46K: 75.2 ± 6.3 AU; *n* = 3; ANOVA *p* < 0.0001; Tukey’s *post hoc*; GST: 6.1 ± 4.4 AU; GST-Syn: 68.0 ± 6.8 AU; GST-A53T: 67.1 ± 3.2 AU; *n* = 5; ANOVA *p* < 0.0001; Tukey’s *post hoc*).

### α-Synuclein does not affect Hsc70-mediated clathrin disassembly *in vitro*

**Figure 5.**
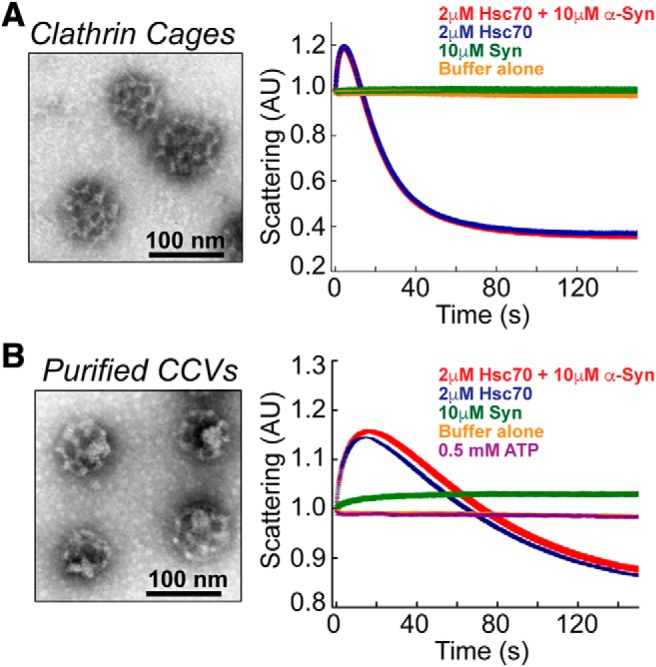
α-Synuclein does not affect Hsc70-mediated clathrin disassembly *in vitro*. ***A***, left, Clathrin cages were assembled *in vitro* using recombinant clathrin heavy chain and auxilin, as described in Sousa et al. (2016). Right, An *in vitro* light scattering assay showed that addition of 2 μM Hsc70 exponentially reduced the light scattering intensity as clathrin cages were disassembled (blue trace). Addition of 10 μM α-synuclein did not alter the rate of Hsc70-mediated clathrin disassembly (red trace). No change in light scattering was observed in baseline control measurements after addition of buffer (yellow trace) or 10 μM α-synuclein alone (green trace). ***B***, left, CCVs, which contain the underlying endocytic vesicle membranes, were freshly purified from bovine brains. Right, α-Synuclein alone slightly increased light scattering (green trace), which is likely due to its binding to the endocytic vesicles. Similar to the results with clathrin cages, introduction of 10 μM α-synuclein did not significantly affect the dynamics of Hsc70-mediated CCV uncoating (blue vs red trace).

Taken together, the above results showing the *in vivo* clathrin uncoating defect and *in vitro* interaction between Hsc70 and α-synuclein suggest that when in excess, α-synuclein may interact with Hsc70 and alter its CCV uncoating function at synapses. Among the possible mechanisms, α-synuclein could interfere with Hsc70 uncoating activity and/or alter Hsc70 availability at synapses. We began testing these possibilities by examining whether α-synuclein affects the ability of Hsc70 to promote clathrin cage disassembly *in vitro* using a light scattering assay as previously described (Sousa et al., 2016). In one set of experiments, we used empty clathrin cages assembled *in vitro* (Fig. 5*A*). In a typical reaction, Hsc70 binds the clathrin cages and subsequently uncoats clathrin, as indicated by a transient increase in light scattering followed by an exponential decrease in scattering intensity as the clathrin is disassembled (Fig. 5*A*, blue trace). Even when α-synuclein was in excess of Hsc70, it had no effect on the disassembly of clathrin cages (Fig. 5*A*, red trace).

It is well established that α-synuclein interacts *in vitro* with small vesicles containing anionic lipids and *in vivo* with synaptic vesicles, and such vesicle interactions promote folding of the NTD into an α-helix (Maroteaux et al., 1988; Davidson et al., 1998; Fortin et al., 2005; Burré et al., 2015). Thus, because α-synuclein effects on uncoating may require membrane binding, we repeated the *in vitro* clathrin uncoating experiments using CCVs purified from bovine brains, which contained the underlying endocytic vesicle membranes (Fig. 5*B*). Under these conditions, some binding of α-synuclein to purified CCVs occurred, as indicated by an initial increase in light scattering (Fig. 5*B*, green trace). As with the clathrin baskets, Hsc70 also bound and disassembled purified CCVs, although the decay in light scattering was less pronounced due to the presence of the vesicular membranes in the reaction (Fig. 5*B*, blue trace). Excess α-synuclein also had no obvious effect on Hsc70-mediated uncoating of purified CCVs (Fig. 5*B*, red trace). Using these highly optimized, reduced conditions, we thus conclude that α-synuclein does not directly interfere with the kinetics or core process of Hsc70-mediated clathrin uncoating *in vitro*.

### Excess α-synuclein inhibits Hsc70 availability at synapses

**Figure 6.**
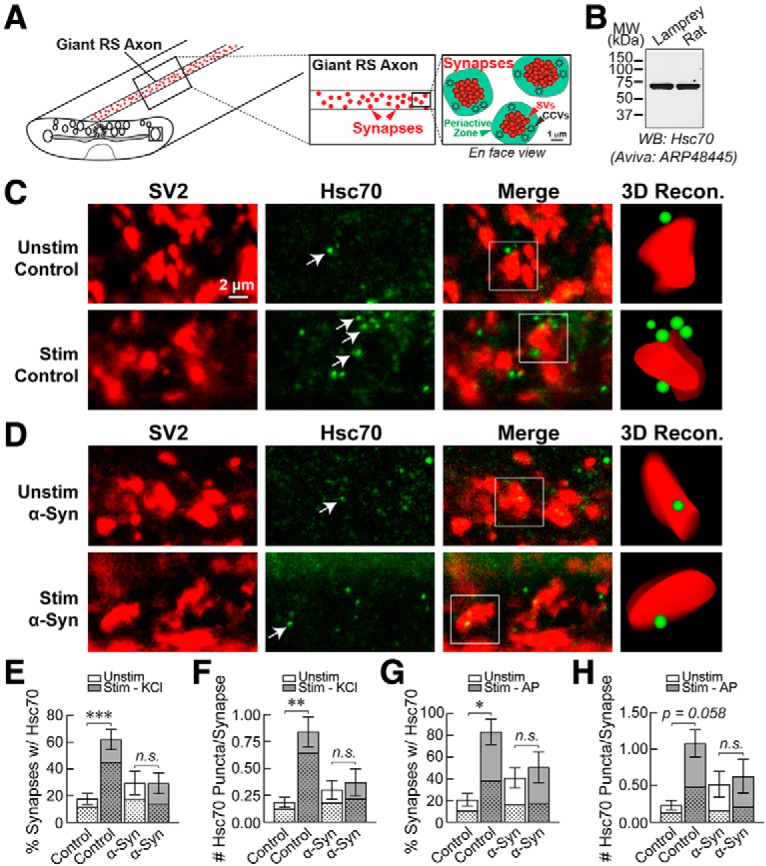
Excess α-synuclein inhibits Hsc70 availability at lamprey synapses. ***A***, Diagram showing the basic organization of the giant RS synapses within lamprey spinal cord. The large synaptic vesicle (SV) clusters (red) are surrounded by a distinct periactive zone where CME occurs (green). ***B***, By Western blotting, the Hsc70 antibody used for these experiments (Aviva ARP48445) specifically recognized a single band at 70 kDa in both lamprey CNS and rat brain lysates, consistent with the expected molecular weight of Hsc70. ***C***, Confocal images showing clusters of giant synapses immunostained for the synaptic vesicle-associated protein SV2 (red) and Hsc70 (green). Compared to unstimulated conditions, stimulation with high K^+^ increased Hsc70 availability at synapses, as evidenced by an increase in the number of visible Hsc70 puncta (arrows). ***D***, In contrast, α-synuclein inhibited the stimulation-dependent increase in Hsc70 at synapses. ***E***, ***F***, Graphs showing the percentage of synapses (per axon) with associated Hsc70 puncta, as well as the average number of Hsc70 puncta per synapse. Bars represent mean ± SEM from *n* = 76–135 synapses, *n* = 5–13 axons, *n* = 5–7 animals/condition; *p < 0.05, **p < 0.01, ***p < 0.001, ****p < 0.0001; n.s. = not significant by ANOVA. ***G***, ***H***, Similar results were obtained using action potential stimulation (20 Hz, 5 min) and a different Hsc70 antibody (Enzo Life Sciences SPA815). Bars represent mean ± SEM from *n* = 31–47 synapses, *n* = 3–4 axons, *n* = 2–3 animals/condition; *p < 0.05, **p < 0.01; n.s. = not significant by ANOVA. In panels ***E–H***, the stippled regions of the bars represent the proportion of Hsc70 puncta overlapping or touching the SV cluster, while the clear regions indicate the proportion localized within the periactive zone.

Compared to the *in vitro* assays, the complex *in vivo* environment of synapses permits a more physiologic partitioning of α-synuclein and Hsc70 between cytosol and synaptic membranes, raising the possibility of observing more functional interactions. We therefore examined whether excess α-synuclein affects the localization of Hsc70 *in vivo* at lamprey synapses. To do so, we performed whole mount immunostaining in the lamprey spinal cord for Hsc70 and SV2, a marker of synaptic vesicle clusters, and then imaged the Hsc70 in the vicinity of the synapses. Giant RS synapses are large *en passant* synapses (1–2 μm in diameter; 1000–2000 synaptic vesicles), which reside along the periphery of the giant RS axons (Fig. 1*B*, inset). They comprise large synaptic vesicle clusters at the active zone, which are surrounded by a distinct actin-rich periactive zone where clathrin-mediated synaptic vesicle recycling occurs (Fig. 6*A*; Shupliakov et al., 2002; Morgan et al., 2004; Bourne et al., 2006; Saheki and De Camilli, 2012). To detect Hsc70, we used an Hsc70 antibody (Aviva ARP48445 rabbit polyclonal) that detects a single 70 kDa band in both lamprey and rat brain lysates by Western blotting (Fig. 6*B*). After immunostaining the spinal cords, the giant RS synapses were first identified using a monoclonal SV2 antibody, which labels presynaptic vesicle clusters in all vertebrates tested, including lampreys (Fig. 6*C*,*D*; Buckley and Kelly, 1985; Busch et al., 2014). The synapse-associated Hsc70 was subsequently evaluated. The Hsc70 antibody immunolabeled distinct puncta within axons and around RS synapses, as observed at mammalian synapses (Wilhelm et al., 2014), as well as diffuse patches (Fig. 6*C*,*D*). Although Hsc70 is one of the most abundant proteins in neurons, the IF signals at both lamprey and mammalian synapses is dimmer than expected, which may be due to some Hsc70 being tightly bound in chaperone protein complexes and therefore inaccessible for immunolabeling. At unstimulated control synapses, the immunolabeled Hsc70 puncta were fairly sparse and in low abundance (Fig. 6*C*, top row). In contrast, stimulation using high K^+^ induced an increase in Hsc70 puncta within or adjacent to the synaptic vesicle clusters, indicating enhanced Hsc70 availability at synapses (Fig. 6*C*, second row). The average size of Hsc70 puncta was 0.20 ± 0.004 μm^2^ (*n* = 174 puncta, *n* = 132 synapses, *n* = 33 axons). Next, as with the EM experiments, we injected human α-synuclein to a final axonal concentration of 7–13 μM (see Materials and Methods). Measurements of endogenous α-synuclein range from 3–6 μM (Westphal and Chandra, 2013) to 45 μM (Wilhelm et al., 2014), suggesting that the total α-synuclein concentration after injection in lamprey axons could be anywhere between 10–58 μM, which is submolar to equimolar with measurements of endogenous Hsc70 in rat synaptosomes (55 μM; Wilhelm et al., 2014). After introducing excess α-synuclein, the stimulation-dependent increase in Hsc70 at presynapses was no longer observed, suggesting an *in vivo* interaction with α-synuclein (Fig. 6*D*). Next, we quantified all of the Hsc70 puncta associated with synapses, both those directly touching or overlapping the synaptic vesicle cluster, as well as those within a 1-μm radius of the synaptic vesicle cluster, representing the endocytic periactive zone (Fig. 6*A*). In Figure 6*E–H*, the stippled regions of the bars indicate the proportion of Hsc70 puncta associated with the synaptic vesicle cluster (30–77% across all conditions), while the unmarked regions indicate the Hsc70 puncta localized exclusively within the periactive zone. At lamprey synapses, many endocytic proteins involved in CME are localized to synaptic vesicle clusters, as well as the periactive zone (Evergren et al., 2004, 2006), and so both pools of Hsc70 may be involved in synaptic vesicle endocytosis. In unstimulated control axons, <20% of synapses per axon had clearly defined immunolabeled Hsc70 puncta associated with them (Fig. 6*E*). Stimulation induced a significant 3-fold increase in the percentage of synapses with Hsc70 puncta, and this did not occur after introduction of excess α-synuclein into the axons (Fig. 6*E*; unstimulated control: 17.61 ± 4.11%, *n* = 85 synapses, *n* = 9 axons; stimulated control: 62.06 ± 7.49%, *n* = 135 synapses, *n* = 13 axons; unstimulated α-Syn: 29.39 ± 8.76%, *n* = 77 synapses, *n* = 5 axons; stimulated α-Syn: 29.26 ± 7.80%, *n* = 76 synapses, *n* = 6 axons; ANOVA *p* < 0.0005; Tukey’s *post hoc*). We also quantified the average number of Hsc70 puncta per synapse under the same conditions and obtained similar results. At control synapses, the average number of Hsc70 puncta per synapse increased on stimulation, but this did not occur after α-synuclein treatment (Fig. 6*F*; unstimulated control: 0.19 ± 0.05 puncta, *n* = 85 synapses, *n* = 9 axons; stimulated control: 0.84 ± 0.14 puncta, *n* = 135 synapses, *n* = 13 axons; unstimulated α-Syn: 0.30 ± 0.09 puncta, *n* = 77 synapses, *n* = 5 axons; stimulated α-Syn: 0.37 ± 0.13 puncta, *n* = 76 synapses, *n* = 6 axons; ANOVA *p* = 0.0012, Tukey’s *post hoc*). In an independent set of experiments, using action potential stimulation (20 Hz, 5 min as in the EM experiments) and a second Hsc70 antibody (Enzo SPA815 rat polyclonal), we observed similar results (Fig. 6*G*,*H*; % synapses w/Hsc70 puncta: unstimulated control: 20.48 ± 5.79%, *n* = 34 synapses, *n* = 3 axons; stimulated control: 83.23 ± 11.75%, = 47 synapses, *n* = 3 axons; unstimulated α-Syn: 40.74 ± 11.75%, *n* = 31 synapses, *n* = 3 axons; stimulated α-Syn: 50.58 ± 14.11%, *n* = 43 synapses, *n* = 4 axons; ANOVA *p* = 0.03; Tukey’s *post hoc*; #Hsc70 puncta/synapse: unstimulated control: 0.23 ± 0.07%, *n* = 34 synapses, *n* = 3 axons; stimulated control: 1.08 ± 0.19%, *n* = 47 synapses, *n* = 3 axons; unstimulated α-Syn: 0.52 ± 0.18%, *n* = 31 synapses, *n* = 3 axons; stimulated α-Syn: 0.63 ± 0.23%, *n* = 43 synapses, *n* = 4 axons; ANOVA *p* = 0.08; Tukey’s *post hoc*). Thus, excess α-synuclein sequesters Hsc70 *in vivo* and reduces its availability at stimulated synapses, suggesting a possible mechanism underlying the CCV uncoating defects during synaptic vesicle recycling.

### Increasing Hsc70 ameliorates the α-synuclein-induced vesicle trafficking defects

**Figure 7.**
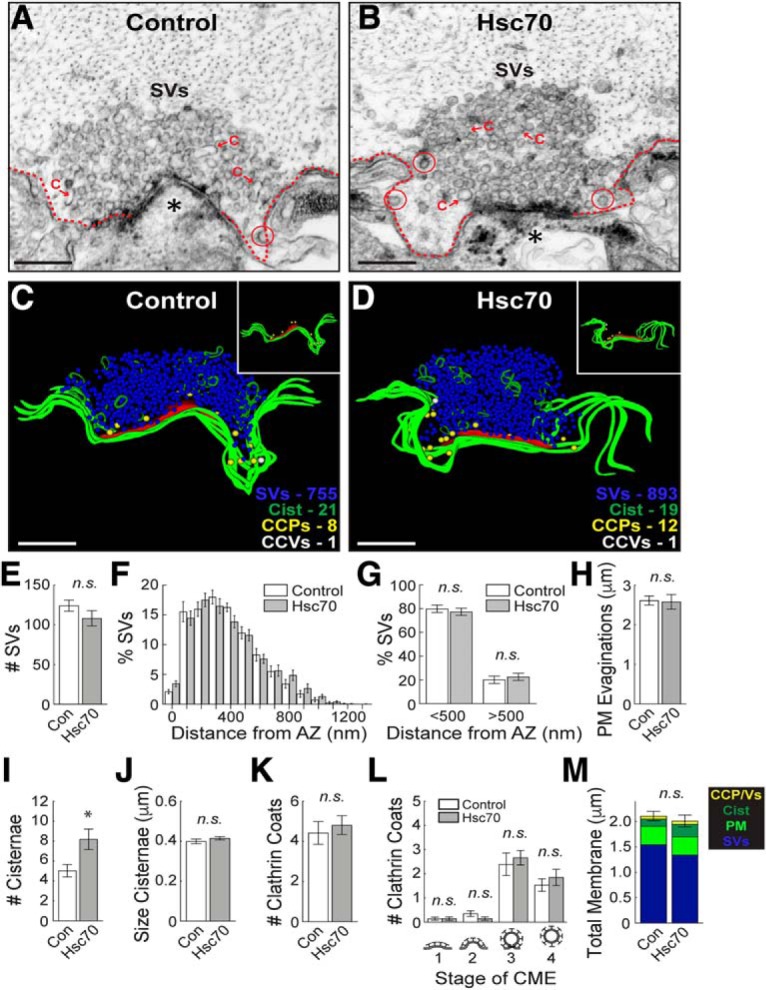
Increasing Hsc70 alone has no effect on CME at synapses. ***A***, ***B***, Hsc70 does not dramatically alter synaptic morphology, as demonstrated by the large synaptic vesicle (SV) clusters, shallow plasma membrane evaginations (dotted lines), and few CCP/Vs (circles) in both control and Hsc70-treated synapses. Asterisks mark postsynaptic spines. C = cisternae. Scale bars = 500 nm. ***C***, ***D***, 3D reconstructions further highlight the similarities and show that CCPs (yellow spheres) and CCVs (white spheres) are in normal numbers and clustered around the plasma membrane (green slabs; see insets). Active zone is shown in red. Scale bars = 500 nm. ***E–M***, There is little effect of exogenous Hsc70 on clathrin-mediated synaptic vesicle endocytosis, as illustrated by normal number and distribution of SVs, PM evaginations, and CCPs/CCVs. Only the number of cisternae was greater, however, their size remains normal. Bars represent mean ± SEM (per section, per synapse) from *n* = 27–28 synapses, *n* = 2 axons, *n* = 2 animals/condition. Asterisk indicates significance (*p* < 0.05); n.s. = not significant (*p* > 0.05) by Student’s *t* test (***E***, ***H–K***, ***M***) or ANOVA (***F***, ***G***, ***L***).

The above results suggest that reduced availability of Hsc70 at synapses, and consequently its function, could underlie the α-synuclein-induced defects in CCV uncoating and synaptic vesicle recycling. If so, then increasing Hsc70 levels would be expected to reverse the synaptic defects. To test this, we co-injected recombinant bovine Hsc70 (1–3 μM final) along with human α-synuclein (7–13 μM final) and examined the effects at lamprey synapses. As a control for these experiments, we first injected Hsc70 alone (Fig. 7). As expected for an abundant chaperone protein under unperturbed conditions, Hsc70 had little effect on synaptic ultrastructure, and the synapses appeared normal (Fig. 7*A-D*). Quantification showed no effect on the synaptic vesicle clusters, plasma membrane, or the number or distribution of CCP/Vs (Fig. 7*E–H*,*K*,*L*; synaptic vesicles %, control: 124.2 ± 7.0 SVs/section, *n* = 27 synapses, *n* = 2 axons; Hsc70: 108.2 ± 9.6 SVs; *n* = 28 synapses, *n* = 2 axons; Student’s *t* test; *p* = 0.18; SV distribution, <500 nm, control: 79.91 ± 3.12%; Hsc70: 77.36 ± 3.02%; >500 nm, control: 20.09 ± 3.12%; Hsc70: 22.64 ± 3.02%; *n* = 27–28 synapses, *n* = 2 axons; ANOVA *p* < 0.0001; Tukey’s *post hoc p* = 0.94; plasma membrane, control: 2.61 ± 0.12 μm, *n* = 27 synapses, *n* = 2 axons; Hsc70: 2.58 ± 0.18 μm; *n* = 28 synapses, *n* = 2 axons; Student’s *t* test; *p* = 0.89; *#*clathrin coats, control: 4.4 ± 0.6 coats, *n* = 27 synapses, *n* = 2 axons; Hsc70: 4.8 ± 0.5 coats, *n* = 28 synapses, *n* = 2 axons; Student’s *t* test; *p* = 0.60; clathrin coat distribution, stage 1, control: 0.14 ± 0.07 CCPs/section; Hsc70: 0.15 ± 0.07 CCPs; stage 2, control: 0.36 ± 0.12 CCPs, Hsc70: 0.15 ± 0.07 CCPs; stage 3, control: 2.39 ± 0.47 CCPs; Hsc70: 2.67 ± 0.30 CCPs; stage 4, control: 1.54 ± 0.26 CCVs, Hsc70: 1.85 ± 0.34 CCVs; *n* = 27–28 synapses, *n* = 2 axons; ANOVA *p* < 0.0001, Tukey’s *post hoc*). The number of cisternae increased slightly, but their size remained unchanged (#cisternae, control: 5.0 ± 0.6 cisternae, *n* = 27 synapses, *n* = 2 axons; Hsc70: 8.2 ± 1.0 cisternae; *n* = 28 synapses, *n* = 2 axons; Student’s *t* test; *p* = 0.01; size cisternae, control: 0.40 ± 0.01 μm, *n* = 141 cisternae, *n* = 27 synapses; Hsc70: 0.42 ± 0.01 μm; *n* = 221 cisternae, *n* = 28 synapses, *n* = 2 axons; Student’s *t* test; *p* = 0.28). Thus, introducing Hsc70 alone produced very little effect on synaptic vesicle trafficking with no measurable effects on CME or synaptic vesicle clusters.

**Figure 8.**
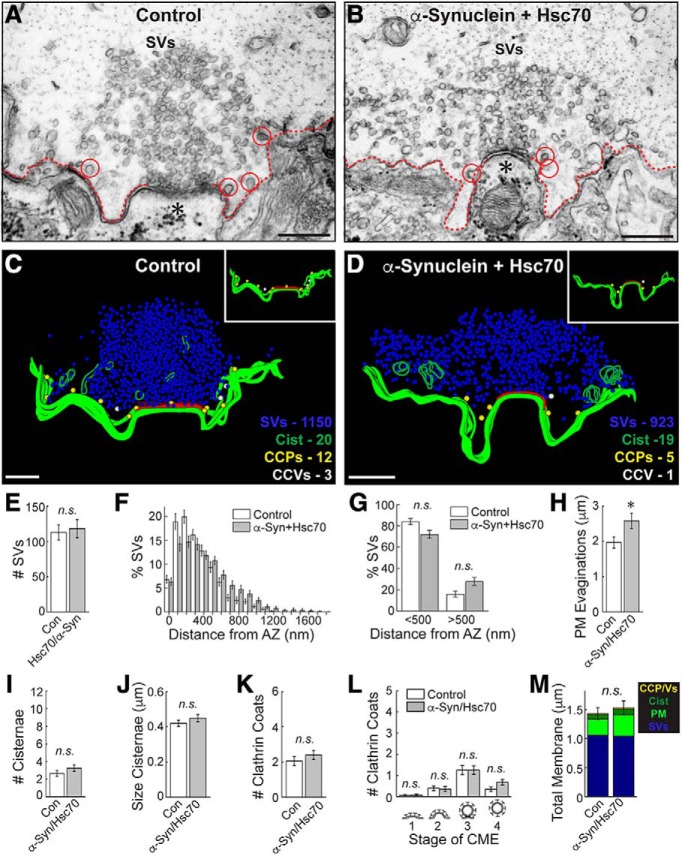
Increasing exogenous Hsc70 largely reverses the α-synuclein-induced synaptic defects. ***A***, ***B***, Unlike synapses treated with α-synuclein alone (Fig. 2), those co-treated with Hsc70 and α-synuclein appear similar to control synapses, with large SV clusters and few cisternae or CCP/Vs (circles). ***C***, ***D***, 3D reconstructions reveal that synapses treated with Hsc70 and α-synuclein appear normal. Insets show the distributions of CCPs (yellow spheres) and CCVs (white spheres), which are sparse and clustered around the plasma membrane (green slabs). Active zone is shown in red. ***E–M***, The CCV uncoating and vesicle recycling defects caused by α-synuclein were largely ameliorated by co-injection of Hsc70 as evidenced by normal numbers of SVs (***E***), cisternae (***J***), and CCPs/CCVs (***K–L***). Only the PM evaginations were larger (***H***). Notably, there was no longer any difference in the number of free CCVs after co-injection of Hsc70+α-synuclein, indicating a reversal of the uncoating defects (***L***, stage 4). Bars represent mean ± SEM (per section, per synapse) from *n* = 22–30 synapses, *n* = 2 axons, 2 *n* = animals/condition. Asterisk indicates significance (*p* < 0.05); n.s. = not significant (*p* > 0.05) by Student’s *t* test (***E***, ***H–K***, ***M***) or ANOVA (***F–G***, ***L***).

We next examined synapses co-treated with both α-synuclein and Hsc70. As before, stimulated control synapses exhibited large vesicle clusters, shallow membrane evaginations, and few CCPs and CCVs (Fig. 8*A*,*C*). Compared to synapses treated with α-synuclein alone, which exhibited dramatic CCV uncoating and synaptic vesicle recycling defects (Fig. 2), those co-treated with α-synuclein and bovine Hsc70 appeared relatively normal with large synaptic vesicle clusters, and only a few cisternae, CCPs, and CCVs (Fig. 8*B*,*D*). 3D reconstructions revealed the remarkable extent to which Hsc70 ameliorated the α-synuclein-associated synaptic vesicle trafficking defects (Fig. 8*C*,*D*). After introducing bovine Hsc70 along with human α-synuclein, the CCPs and CCVs were now sparse and located near the plasma membrane, similar to their distribution at control synapses (Fig. 8*C*,*D*, insets). Quantitative analyses revealed no difference in the number of synaptic vesicles at synapses co-treated with Hsc70+α-synuclein versus controls (Fig. 8*E*; control: 105 ± 11 SVs/section; Hsc70+α-Syn: 113 ± 13 SVs/section; *n* = 22–30 synapses, *n* = 2 axons; Students *t* test; *p* = 0.644). Although the SVs still appeared slightly dispersed (Fig. 8*F*), the percentages of vesicles near to (<500 nm) and far from (>500 nm) the active zone were no longer significantly different (Fig. 8*G*; <500 nm, control: 84.03 ± 2.82%; α-Syn+Hsc70: 72.07 ± 3.71%; >500 nm, control: 15.97 ± 2.82%; α-Syn+Hsc70: 27.93 ± 3.71%; *n* = 20–28 synapses, *n* = 2 axons/condition; ANOVA *p* < 0.0001; Tukey’s *post hoc p* = 0.09). The synaptic vesicles were of similar size (diameter) in both conditions (control: 54.6 ± 0.6 nm, *n* = 200 SVs, 10 synapses; Hsc70+α-Syn: 52.9 ± 0.8 nm; *n* = 200 SVs, *n* = 10 synapses; Student’s *t* test; *p* = 0.07). Similarly, the number and size of cisternae were unchanged (Fig. 8*I*,*J*; #cisternae, control: 2.7 ± 0.3 cisternae/section; Hsc70+α-Syn: 3.3 ± 0.4 cisternae/section; *n* = 20–28 synapses, *n* = 2 axons; Student’s *t* test; *p* = 0.26; size cisternae, control: 0.42 ± 0.02 μm; Hsc70+α-Syn: 0.45 ± 0.02 μm; *n* = 53–91 cisternae, *n* = 20–28 synapses; Student’s *t* test; *p* = 0.34). Hsc70 additionally restored the total number of clathrin-coated structures (CCPs+CCVs) to control levels (Fig. 8*K*; control: 2.1 ± 0.3 coats/section; Hsc70+α-Syn: 2.4 ± 0.2 coats/section; *n* = 20–28 synapses, *n* = 2 axons; Student’s *t* test; *p* = 0.35). Furthermore, there was a complete rescue of the CCV uncoating defect (Fig. 8*L*; stage 1, control: 0.05 ± 0.05 CCPs; Hsc70+α-Syn: 0.07 ± 0.05 CCPs; stage 2, control: 0.40 ± 0.11 CCPs; Hsc70+α-Syn: 0.36 ± 0.12 CCPs; stage 3, control 1.25 ± 0.22 CCPs, Hsc70+α-Syn: 1.25 ± 0.20 CCPs; stage 4, control: 0.35 ± 0.11 CCVs, Hsc70+α-Syn: 0.68 ± 0.14 CCVs; *n* = 20–28 synapses; ANOVA *p* = 3.9 × 10^−12^, Tukey’s *post hoc*). Only the plasma membrane evaginations remained larger after Hsc70 and α-synuclein co-injection, indicating some persistent effects on the plasma membrane (Fig. 8*H*; control: 1.95 ± 0.15 μm/section; Hsc70+α-Syn: 2.53 ± 0.20 μm; *n* = 22–30 synapses; Student’s *t* test; *p* = 0.04). The total membrane analysis further corroborated that synapses co-treated with Hsc70 and α-synuclein were similar to controls (Fig. 8*M*; control 1.4 ± 0.1 μm^2^; α-Syn: 1.5 ± 0.1 μm^2^; *n* = 20–28 synapses, *n* = 2 axons; Student’s *t* test; *p* = 0.70). Thus, increasing exogenous Hsc70 levels largely reversed the synaptic vesicle trafficking defects caused by α-synuclein, including the deficits in CCV uncoating.

## Discussion

**Figure 9.**
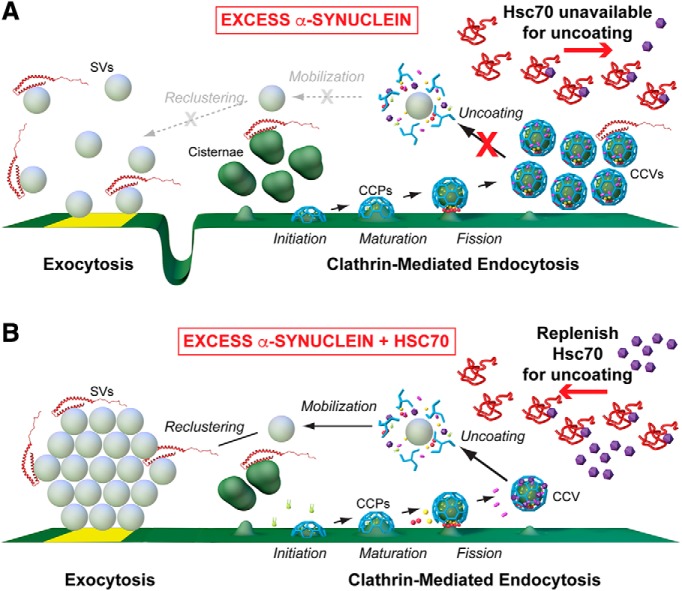
Working model for α-synuclein-induced endocytic defects and amelioration by Hsc70. ***A***, In the presence of excess α-synuclein, endogenous Hsc70 becomes depleted at synapses, leading to impaired CCV uncoating and an inhibition of synaptic vesicle recycling. ***B***, Addition of exogenous Hsc70 restores CCV uncoating and leads to more normal synaptic vesicle recycling. (Graphics by Jack Cook and Tim Silva, Woods Hole Oceanographic Institution.)

While a number of studies have focused on the physiologic roles of α-synuclein at synapses (for review, see Sulzer and Edwards, 2019), the precise effects of excess α-synuclein and the underlying mechanisms are less clear. Data presented here identify loss of Hsc70 availability at synapses, and consequently its function, as one mechanism by which excess α-synuclein induces synaptic vesicle trafficking defects. Specifically, the sequestration of Hsc70 leads to an impairment of CCV uncoating at synapses, which consequently inhibits synaptic vesicle recycling. How might this work? A plausible explanation, shown in our working model, is that inhibiting CCV uncoating may trap clathrin and/or other limited coat proteins such as AP180 and AP2 within CCVs, making them unavailable for initiating subsequent rounds of endocytosis thereby leading to aberrant plasma membrane expansion and compensatory bulk endocytosis, resulting in formation of cisternae (Fig. 9*A*; Morgan et al., 1999, 2000; Walsh et al., 2018). Similarly, acute inactivation of CME in other models also resulted in a compensatory increase in atypical cisternae (Heerssen et al., 2008; Kasprowicz et al., 2008). In these respects, the phenotypes produced by excess α-synuclein are suggestive of a pathologic gain of function. However, the plasma membrane expansion observed with excess α-synuclein would also be consistent with direct effects of α-synuclein accelerating vesicle fusion (Logan et al., 2017) and/or slowing early stages of clathrin-mediated synaptic vesicle endocytosis (Vargas et al., 2014), which is in line with our current understanding of its normal physiologic function. Similar to the α-synuclein phenotype, impairments of CCV uncoating have also been observed at squid and mammalian synapses after directly perturbing Hsc70 recruitment to CCVs (Morgan et al., 2001; Leshchyns’ka et al., 2006). As with direct Hsc70 perturbations (Morgan et al., 2001), excess α-synuclein also induced a loss of synaptic vesicles and expansion of the plasma membrane, indicating effects on vesicle recycling (Fig. 2; Busch et al., 2014; Medeiros et al., 2017).

By this working model, replenishing Hsc70 at synapses should have the effect of restoring CCV uncoating, thereby freeing clathrin and other limited coat proteins to recycle synaptic vesicles more efficiently (Fig. 9*B*). Indeed, when exogenous bovine Hsc70 was introduced along with human α-synuclein, the CCV uncoating defects were completely rescued, and the synaptic vesicle clusters were restored to normal, indicating a significant improvement in synaptic vesicle recycling (Fig. 8). That the number and size of cisternae were also restored to control levels indicates their role in replenishing the vesicle cluster and suggests that they are affected by clathrin-dependent budding processes, although their identity remains unclear (e.g., recycling or bulk endosomes). The only morphologic deficits remaining after co-injection of α-synuclein and Hsc70 was an enlargement of the plasma membrane evaginations and a slight, but non-significant, dispersion of synaptic vesicles (Fig. 8). One possible explanation is that there may be a pool of α-synuclein bound tightly to the plasma membrane and vesicles, which does not release in the presence of exogenous Hsc70 and therefore continues to slow endocytosis. While our data are consistent with the working model shown in Figure 9, we also acknowledge the possibility of an alternative explanation that α-synuclein may inhibit vesicle recycling by some other undetermined mechanism, which is reversed by Hsc70 for example through its general chaperoning functions. Whatever the case, it is an exciting prospect that even substoichiometric amounts of Hsc70 can reverse the vast majority of the synaptic vesicle trafficking defects associated with excess α-synuclein. It will be important in future experiments to determine if the remaining morphologic alterations can be further ameliorated by increasing the concentration of co-injected Hsc70 or by introducing Hsp110, which was recently shown to mitigate α-synuclein pathology in mouse models (Taguchi et al., 2019) and is the most likely Hsc70 nucleotide exchange factor that regulates availability of free clathrin at synapses (Morgan et al., 2013).

Our results corroborate and extend previous reports of an interaction between α-synuclein and Hsc70 (Pemberton et al., 2011; Redeker et al., 2012). Hsc70 binds both soluble and fibrillar α-synuclein in the absence of nucleotides and inhibits fibril formation *in vitro*, leading to increased cell viability (Pemberton et al., 2011). Cross-linking studies using soluble α-synuclein and Hsc70 indicated two discrete regions in α-synuclein (a.a. 10–45, 97–102) that bind to multiple sites within the client binding domain of Hsc70, including several in the C-terminal region (Redeker et al., 2012). Similar binding sites were observed between α-synuclein and the yeast Hsc70 ortholog, Ssa1p, indicating conservation of the interaction (Redeker et al., 2012). Our results further demonstrate conservation across synuclein orthologs (i.e., lamprey and human), which is due to the high degree of amino acid identity shared between their NTDs (Busch and Morgan, 2012). In the first Hsc70 binding region of α-synuclein (a.a.10–45), lamprey γ-synuclein is 78% identical and 89% similar to human α-synuclein (Busch et al., 2014). The second Hsc70 binding region (a.a. 97–102) is less conserved in lamprey γ-synuclein with only 30% similarity to human α-synuclein, suggesting that the longer N-terminal binding region (a.a. 10–45) is the stronger of the two binding regions. Wild-type α-synuclein, A30P, and A53T all bound with equal efficacy to Hsc70 (Fig. 4) likely because the binding is distributed across the NTD region of α-synuclein (Redeker et al., 2012). In contrast, binding of Hsc70 to E46K was reduced, perhaps because this mutation alters the charge distribution around the Hsc70 binding site. We also show that the C-terminal region of Hsc70 is critical for mediating and/or stabilizing the interaction with α-synuclein. The failure of Hsc70ΔC to bind α-synuclein is consistent with observations that the region deleted in Hsc70ΔC includes residues that form part of the interface with α-synuclein (Redeker et al., 2012). In addition, the deletion in Hsc70ΔC destabilizes the terminal helix of Hsc70 SBD, resulting in unwinding of this helix and cis-binding of its unfolded segment in the client binding site of nucleotide free Hsc70 (Morshauser et al., 1999; Jiang et al., 2005). The client binding site in Hsc70 also contributes to the interface with α-synuclein (Redeker et al., 2012), and so cis-binding of this unfolded segment may compete with α-synuclein binding. Going forward, it will be important to identify the most critical residues mediating the α-synuclein/Hsc70 interaction to facilitate the design of reagents that disrupt this interaction, which might have therapeutic value for treatment of multiple diseases associated with α-synuclein and/or clathrin uncoating defects.

Hsc70 was previously identified as a potential target in other α-synuclein-related contexts. In human brains affected by PD, DLB, and related synucleinopathies, Hsc70 and other chaperone proteins are detected in high levels along with α-synuclein (Auluck et al., 2002; Uryu et al., 2006). The co-occurrence of Hsc70 and α-synuclein in Lewy bodies suggests an increased association of these proteins in disease states, which could deplete Hsc70 and impair its function in other cellular compartments. In support of this idea, α-synuclein aggregation, fibrillation, trans-synaptic propagation, and neurotoxicity are reduced in the presence of Hsc70 or Hsp70 (Luk et al., 2008; Danzer et al., 2011; Pemberton et al., 2011). Furthermore, overexpression of Hsp70 reduced α-synuclein aggregation and neuronal loss in *Drosophila* and mouse models of α-synuclein toxicity (Auluck et al., 2002; Klucken et al., 2004). Thus, Hsc/p70-based reagents that can reverse misfolding and/or restore normal protein function have been suggested as potential therapeutic agents to reduce neurodegeneration caused by α-synuclein (Dimant et al., 2012; Ebrahimi-Fakhari et al., 2013; Shorter, 2016). Our data extend the possible applications of such agents by showing that replenishing Hsc70 function may also be a viable strategy for improving the synapse-associated deficits observed in α-synuclein-related disorders.

Importantly, our work also provides new insights into the cellular mechanisms for α-synuclein and Hsc70 involvement in the neurodegenerative process, specifically related to the impacts on CME. Since CME is also involved more broadly in vesicle endocytosis from the plasma membrane and intracellular vesicle trafficking events, these experiments also have wider implications for potential impacts on membrane trafficking throughout the entire neuron. In addition, our work also highlights the need for the type of studies described here, in which the mechanism of phenotypic rescue is characterized in depth, because of the potential complexities of deploying a therapeutic approach that involves increasing the activity of a protein like Hsc70, which is involved in many cellular processes. We find that while Hsc70 fully rescues the CCV uncoating defects induced by α-synuclein and restored the synaptic vesicle cluster, the membrane evaginations persisted, suggesting residual impairment of membrane endocytosis by α-synuclein (Vargas et al., 2014). Such observations suggest not only the need to titrate such therapeutic approaches, but also to understand the mechanisms of phenotypic rescue so that any complications can be anticipated.

The findings reported here contribute to a growing body of evidence that PD and other forms of Parkinsonism may be linked to deficits in the clathrin-mediated synaptic vesicle recycling (Saez-Atienzar and Singleton, 2017), and an increasing number of findings are pointing toward specific impairments in the clathrin uncoating process. For example, many studies have now identified mutations or truncations in *DNAJ*, the gene that encodes for the Hsc70 co-chaperone, auxilin, in patients with juvenile Parkinsonism or early-onset PD (Edvardson et al., 2012; Köroğlu et al., 2013; Elsayed et al., 2016; Olgiati et al., 2016). In addition, auxilin was recently identified as a phosphorylation target of the PD-linked leucine-rich repeat kinase 2 (LRRK2) mutant R1441C, which led to impaired SV endocytosis and clathrin uncoating defects in patient-derived dopaminergic neurons (Nguyen and Krainc, 2018). Furthermore, GWAS studies have identified genetic variations and altered expression of GAK, the ubiquitously expressed version of neuronal-specific auxilin, among the top risk factors for familial PD across multiple populations worldwide (Pankratz et al., 2009; Tseng et al., 2013; Nagle et al., 2016). Several mutations in synaptojanin-1 have also been linked to early onset Parkinsonism (Krebs et al., 2013; Chen et al., 2015; Ben Romdhan et al., 2018). Transgenic mice carrying one of the synaptojanin-1 mutations (R258Q) in the Sac domain accumulated CCVs within their synapses and exhibited impaired synaptic vesicle recycling, as well as motor deficits and increased death (Cao et al., 2017). Thus, a growing body of evidence from both animal models and human genetics indicates that defects in CME, and specifically in CCV uncoating, are at least susceptibility factors in PD and Parkinsonism, if not causal factors. Detection of mutations in auxilin that increase such susceptibility is especially notable because, unlike Hsc70 or other chaperones that have multiple cellular functions, auxilin’s role appears to be limited to the single function of recruiting Hsc70 to CCVs to drive uncoating, so these observations strongly indicate that disruption of clathrin uncoating is a strong contributor to PD disease etiology. Thus, strategies that ensure proper CCV uncoating, for example by increasing Hsc70 availability or function, may hold promise for improving synaptic function and reducing neurodegeneration in PD and other α-synuclein-associated diseases.

## Synthesis

Reviewing Editor: Pablo Castillo, Albert Einstein College of Medicine Decisions are customarily a result of the Reviewing Editor and the peer reviewers coming together and discussing their recommendations until a consensus is reached. When revisions are invited, a fact-based synthesis statement explaining their decision and outlining what is needed to prepare a revision will be listed below. The following reviewer(s) agreed to reveal their identity: NONE. Note: If this manuscript was transferred from JNeurosci and a decision was made to accept the manuscript without peer review, a brief statement to this effect will instead be what is listed below.

Your revised manuscript and rebuttal letter was evaluated by an expert in the field. This reviewer understands that your manuscript provides a specific and new model for explaining the inhibitory effect of alpha-synuclein on synaptic vesicle recycling and endocytosis. For the most part, you have properly addressed previous criticisms, and your model is certainly possible and sits well with your data, but other interpretations are possible. Among other comments, he/she suggests you should demonstrate that co-injection of hsc70 indeed results in rescue of the increase in the hsc70 puncta. The reviewer is troubled about not knowing whether these puncta actually mean something functional.Below please find his/her specific comments.

----------------------------------------------------------------------------

In this interesting and important manuscript, Banks et al show that injection of human alpha-synuclein (a-syn) produces a pronounced effect on the structure of synaptic contacts in stimulated lamprey giant axons, in agreement with previous work from the same group. They report an increase in the length of membrane evaginations, a decrease in the number of synaptic vesicles, an increase in the number and size of cisternae as well as an increase in the number of clathrin coated structures. They expand previous reports in showing that out of the clathrin coated structures, it is specifically the number of coated vesicles (stage 4) that is increased. Also, they show that the remaining synaptic vesicles are more widely distributed within the peri-active site region. These results are consistent with a deficit in endocytosis, and specifically with a deficit in late stages of the endocytotic process (vesicle uncoating). Still, some elements, like the enhanced number and size of evaginations and cisternae, could indicate additional deficits either in early stages of endocytosis (see Vargas et al. 2014) or an enhancement of exocytosis (or both).

Because they suspected effects on late stages of endocytosis, the authors examined the association of a-syn with Hsc70 (an association that has been reported in other experimental systems). Using pulldown experiments, they show that human a-syn as well as lamprey gamma-syn (which is quite homologous with human a-syn) as well as some of their truncations (their c-terminal removed) interact with Hsc70. No interaction is observed with key proteins involved in other stages of endocytosis. They further report that the a-syn/hsc70 association occurs through the c-terminal region of Hsc70.

The authors further present evidenced (collected by measuring light scattering during uncoating of purified coated vesicles) that a-syn does not affect the kinetics of uncoating by Hsc70.

Importantly for their conclusion, the authors show by light microscopy that stimulation increases the number of Hsc70 puncta observed near synaptic vesicle clusters (stained with SV2), but that this increase is annulled by the injections of a-syn.

Finally, the authors show that injection of Hsc70 on its own has little effect on synaptic morphology (only the number of cisternae was enhanced out of all other parameters), but that co-injection of Hsc70 along with human a-syn ameliorates the effect of a-syn alone (except for the length of the evaginations).

The authors interpret their results as follows: the effect of a-syn on endocytosis, which is similar to the effect of removing Hsc70 in other systems (squid, mammal), is achieved by quantitatively sequestering Hsc70, which is required for the uncoating of synaptic vesicles. They also suggest that the deficit in uncoating decreases in a secondary manner the availability of other key factors required for earlier stages of endocytosis (because they remain linked to the CCVs). Because co-injection of Hsc70 does not fully rescue the synaptic structure, the authors say that they cannot rule out a parallel direct effect of a-syn on earlier stages of endocytosis.

This manuscript is of significant interest because it provides a plausible explanation for the inhibitory effect of a-syn on synaptic vesicle recycling and synaptic activity in general.

This could be of significance in the future for the development of interventions in a-syn deleterious effects.

Review

In my opinion, the authors have performed a laudable job in addressing the many concerns of both reviewers.

However, following are a few issues that need to be addressed and which were not raised previously.

In my opinion, the authors cannot exclude an alternative interpretation of their results regarding the co-injection of a-syn and Hsc70. It appears to me that it is just as probable that Hsc70 sequesters (or in some way inactivates) a-syn [according to the concentrations cited by the authors, the final concentration after injection is 56-58 micromolar for Hsc70 and 10-58 micromolar for a-syn]. According to the alternative model, instead of Hsc70 being the limiting key factor that a-syn sequesters, it is rather Hsc70 that sequesters a-syn. In this model, a-syn inhibits synaptic vesicle endocytosis in an as yet unidentified manner. When excess Hsc70 is introduced, the remaining unsequestered a-syn does not suffice to affect endocytosis. Because the equilibrium constant for the association of the two proteins is unknown, and because the concentration of endogenous a-syn and Hsc70 in lamprey axons is not reported (only in mammalian systems), it is not possible to directly differentiate between these two possibilities.

I see two ways to overcome this issue. The first is to acknowledge the possibility of an alternative explanation in the manuscript. Another is to strengthen the case in favor of the authors' model by additional experimental work similar to that shown in Fig. 6. In this experiment, both exogenous Hsc70 and a-syn are injected; then the number of Hsc70 puncta are quantified, and it is determined whether exogenous Hsc70 indeed rescues the formation of puncta during stimulation, as would be predicted by the authors' model. If the authors choose to address the issue experimentally, then sufficient time should be allowed.

Of course, I am open for the authors to convincingly rule out the alternative model in any other way.

Notwithstanding, I have to point out a weakness in figure 6 - the identity or function of the Hsc70 puncta seen in the fluorescence images is unknown. The authors assume that the puncta relate to a functional cellular structure, but that claim is not substantiated. Furthermore, the resolution limitations of light microscopy invalidate the authors' assertions concerning the size of the Hsc70 puncta. The "size" that the authors state for the puncta (200 micrometers) is in the range of what would be expected to be the size of the image of a resolution-limited point, based on the numerical aperture of the objective and the emission wavelength of the fluorescent dye. Therefore, the authors should not compare its size to that of single CCVs, which are much smaller than the resolution limit.

In future studies the authors may want to validate their results by depleting Hsc70 using a different approach (antibodies? introduction of a different Hsc70 binding protein?). Alternatively, they could inject factors interfering with a-syn/hsc70 binding like interfering peptides or perhaps a C terminal segment of Hsc70 (as was done previously in squid by the senior author). I realize this is outside the scope of this study.

10.1074/jbc.RA118.006551

10.1073/pnas.1903049116

10.1073/pnas.1903054116

10.1111/jnc.14810

10.1073/pnas.1903268116
